# Identification and Expression of the Family of Classical Protein-Tyrosine Phosphatases in Zebrafish

**DOI:** 10.1371/journal.pone.0012573

**Published:** 2010-09-03

**Authors:** Mark van Eekelen, John Overvoorde, Carina van Rooijen, Jeroen den Hertog

**Affiliations:** 1 Hubrecht Institute, KNAW and University Medical Center Utrecht, Utrecht, The Netherlands; 2 Institute of Biology Leiden, Leiden University, Leiden, The Netherlands; Ecole Normale Supérieure de Lyon, France

## Abstract

Protein-tyrosine phosphatases (PTPs) have an important role in cell survival, differentiation, proliferation, migration and other cellular processes in conjunction with protein-tyrosine kinases. Still relatively little is known about the function of PTPs *in vivo*. We set out to systematically identify all classical PTPs in the zebrafish genome and characterize their expression patterns during zebrafish development. We identified 48 PTP genes in the zebrafish genome by BLASTing of human PTP sequences. We verified all *in silico* hits by sequencing and established the spatio-temporal expression patterns of all PTPs by *in situ* hybridization of zebrafish embryos at six distinct developmental stages. The zebrafish genome encodes 48 PTP genes. 14 human orthologs are duplicated in the zebrafish genome and 3 human orthologs were not identified. Based on sequence conservation, most zebrafish orthologues of human PTP genes were readily assigned. Interestingly, the duplicated form of *ptpn23*, a catalytically inactive PTP, has lost its PTP domain, indicating that PTP activity is not required for its function, or that *ptpn23b* has lost its PTP domain in the course of evolution. All 48 PTPs are expressed in zebrafish embryos. Most PTPs are maternally provided and are broadly expressed early on. PTP expression becomes progressively restricted during development. Interestingly, some duplicated genes retained their expression pattern, whereas expression of other duplicated genes was distinct or even mutually exclusive, suggesting that the function of the latter PTPs has diverged. In conclusion, we have identified all members of the family of classical PTPs in the zebrafish genome and established their expression patterns. This is the first time the expression patterns of all members of the large family of PTP genes have been established in a vertebrate. Our results provide the first step towards elucidation of the function of the family of classical PTPs.

## Introduction

Protein-tyrosine phosphatases (PTPs) dephosphorylate phosphotyrosyl residues in proteins that are phosphorylated by protein-tyrosine kinases (PTKs). PTPs and PTKs play an important role in relaying signals in the cell and are tightly regulated [1], [2], [3]. Tyrosine phosphorylation signaling has been shown to control many fundamental processes in the cell and disruption of the balance between phosphorylation and dephosphorylation has been shown to be at the basis of several human diseases [4], [5], [6], [7], [8]. Historically, most signal transduction research has been done on kinases, and the function of most phosphatases remains to be determined.

PTPs in the human genome have been grouped in four classes (I, II, III and IV) based on their catalytic site and substrate specificity [4]. Class I contains the classical PTPs and the dual specificity phosphatases (DUSPs). The classical PTPs can be further subdivided in receptor-like (RPTP) and non-receptor-like (NRPTP) PTPs. The vertebrate genome encodes 37 different classical PTPs. Rodent genomes contain an additional Type 3 receptor gene, *ptprv*, resulting in a total of 38 PTP genes in rodents. The zebrafish is widely used as a model system for developmental biology [9], genetics [10], [11], [12], [13], cancer research [14], [15] and small molecule screens [16]. The zebrafish genome is being sequenced and with the release of Zv8 [17], the zebrafish genome assembly is almost complete. Currently, the zebrafish genome contains 21 genes that are annotated as PTP. We and others have established that PTPs have essential roles in zebrafish embryonic development by analysis of phenotypical defects upon knockdown of target PTP expression, including RPTPα [18], [19], PTPψ [20], Shp2 [21] and PEZ [22]. Given the number of PTPs in other vertebrate genomes, it is most likely that not all PTPs have been identified or annotated as PTP in the zebrafish genome.

In order to start to elucidate the role of classical PTPs, we identified all classical PTPs in the zebrafish genome and established their expression pattern by *in situ* hybridization. Based on homology to human orthologs, we identified 48 classical PTPs in the zebrafish genome. 14 genes are duplicated in zebrafish, compared to human and 3 human orthologs were not identified. Expression of all the genes that we had identified *in silico* was verified by sequencing of fragments of cDNAs that we obtained by reverse transcription-PCR (RT-PCR). Here, we report the expression patterns of all 48 PTP genes that we identified by *in situ* hybridization at six stages of zebrafish development. We focused on the expression patterns of duplicated genes and found that whereas some duplicated genes retained their expression pattern, others have diverging expression patterns. Our results suggest that the function of some duplicated PTP genes is overlapping and the function of others is distinct.

## Results and Discussion

### Identification of all PTPs in the zebrafish genome

Some genes encoding PTPs are annotated in the zebrafish genome but many are conspicuously missing. We used the PTPs found in the human genome as a template in an attempt to systematically identify the entire family of zebrafish PTPs. We used the PTP domain of human proteins which was identified by scanning the peptide sequence on ExPASy Prosite (http://www.expasy.org/prosite/). Whenever a tandem PTP domain was present, the D1 PTP domain was used for BLAST searches. All human PTP domains were BLASTed against the Zv8 version of the zebrafish genome (TBLASTN) and all hits were scanned for PTP domains. We analyzed the hits for the presence of known coding sequences or annotated genes, or when not present for predicted gene sequences (Genscan). All these sequences were subsequently scanned for the presence of PTP domains using Prosite. We used annotated gene sequences or known coding sequences where possible. In other cases, predicted transcripts were used. For some genes (*ptpn3*, *ptpn20*, *ptprt*, *ptprua*, *ptprub* and *ptprjb*) partial known coding sequences were available but they did not cover the PTP domain. In those cases predicted sequences were used for alignment purposes. Yet, known coding sequences were used for sequencing and probe generation. In two cases no known coding sequence was available at all (*ptprm*, *ptprr*). In those cases predicted sequences were used for alignment, probe generation and sequencing.

**Figure 1:**
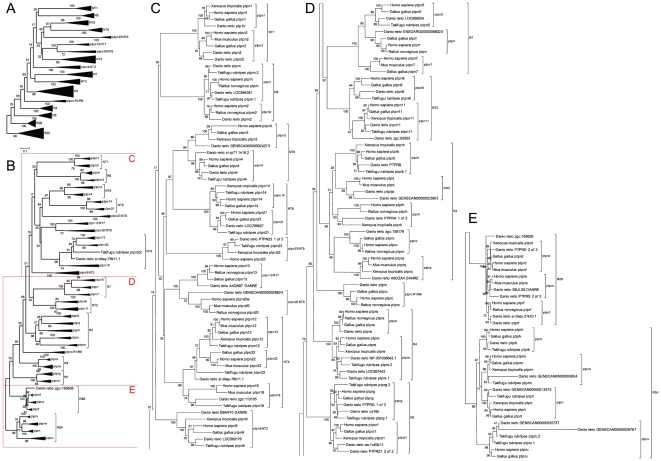
Alignment of vertebrate PTP domains. Protein sequences were obtained from Ensembl database of all PTPs from zebrafish, *Fugu*, *Xenopus*, chicken, mouse, rat and human. The PTP domains were identified using http://www.expasy.org/prosite/ and used for alignment using the MEGA4 program. When a tandem PTP domain was present, the D1 PTP domain was used for the alignment. The evolutionary history was inferred using the Neighbor-Joining method. The optimal tree with the sum of branch length = 31.88069250 is shown. The percentage of replicate trees in which the associated taxa clustered together in the bootstrap test (500 replicates) are shown next to the branches. The tree is drawn to scale, with branch lengths in the same units as those of the evolutionary distances used to infer the phylogenetic tree. The evolutionary distances were computed using the Dayhoff matrix based method and are in the units of the number of amino acid substitutions per site. All positions containing alignment gaps and missing data were eliminated only in pairwise sequence comparisons (Pairwise deletion option). There were a total of 475 positions in the final dataset (Tamura et al. 2007). An overview of the entire collapsed tree is shown in Figure 1A, with individual pieces split to make up figures 1B–E, as indicated. Not all known annotated genes of species other than zebrafish are included.

To verify our hits, we aligned all the protein sequences of PTP domains identified in this way with human, mouse, rat, chicken, *Xenopus* and *fugu* PTP domains obtained from the Ensembl database (Fig. 1). We used the PTP domains identified using Prosite and aligned those sequences with Mega4 software, using the Dayhoff matrix and pairwise deletion. All identified zebrafish PTP genes are listed in Tables 1 and 2, together with Zv8 identifiers. For zebrafish PTP gene and protein names we used conventions as mentioned in [23]. Names were appended with “a” or “b” for duplicates where necessary. Whereas the cladogram in Fig. 1 is based on alignment of proteins, we used gene names for clarity. We identified 48 PTP genes in the zebrafish genome. All of them cluster together with orthologs of other vertebrate species. 14 genes constituted a duplicated gene (28 genes in total) and 3 human orthologs were not identified (*ptpn7*, *ptpn12* and *ptpn14*). The missing PTP genes may have been lost in evolution or these genes may (partially) be located in poorly sequenced areas of the genome, thus preventing identification by BLAST searches. One of these genes, *ptpn14*, encoding Pez has been described previously in zebrafish [22]. The EST sequence at the basis of these experiments is BQ285767.1 which corresponds to LOC799627 in the Ensembl database. Alignment of the PTP domain encoded by LOC799627 indicated more homology to the structurally related *ptpn21* than to *ptpn14* (Fig. **1** **). Possibly there is only one ortholog of the highly related** ***ptpn14*** **and** ***ptpn21*** **in zebrafish.** In our *in silico* screen we found two candidates for zebrafish *ptpn23*, one annotated as *ptpn23* and one annotated as si:dkeyp-114f9.2 in the Ensembl database. It is noteworthy that *Takifugu rubripes* and other fish species have only one copy of the *ptpn23* gene. Si:dkeyp-114f9.2, which does not encode a PTP domain, was designated *ptpn23b*, based on structural and sequence similarity of the BRO domain. BLASTing the coding region of *ptpn23b* to the human genome yields *ptpn23* as the top hit. We cannot exclude the possibility that the PTP domain was not detected because of errors in the current assembly of the zebrafish genome. However, this seems unlikely since there are no gaps present in the region and the sequence quality of the region is good. We did not detect exons encoding a PTP domain between si:dkeyp-114f9.2 (*ptpn23b*) and its flanking gene to the 3′ side (si:dkeyp-114f9.4 / CSPG5). We analyzed the presence of expressed sequence tags (ESTs) to the 3′-side of si:dkeyp-114f9.2 (*ptpn23b*). We found 2 ESTs in this short region, both containing non-coding sequence, likely making them part of the 3′ UTR of *ptpn23b*. We amplified the 3′ end of si:dkeyp-114f9.2 (*ptpn23b*) by reverse transcription PCR, sequenced this area and verified the presence of the stop codon and 3′ UTR as annotated in Ensembl database (EBI identifier: FR668536). Therefore, we conclude that *ptpn23b* does not encode a PTP domain. It is noteworthy that human *ptpn23* encodes a PTP domain that harbors no catalytic activity and functional assays indicated that the function of the protein product of *ptpn23*, HD-PTP, in cell signaling is independent of PTP activity [24]. Yet, deletion of the PTP domain of rat PTP-TD14, encoded by *ptpn23*, abolished its capacity to inhibit Ha-*ras*-mediated focus formation of NIH3T3 cells [25], indicating that whereas PTP-TD14 does not encode an active PTP, its PTP domain is functional. These functional data on human and rat HD-PTP support the hypothesis that *ptpn23* was duplicated and that *ptpn23b* lost its PTP domain in the course of evolution. Whether *ptpn23b* encodes a functional gene remains to be determined.

**Table 1:** Classical non-receptor PTP genes in the zebrafish genome.

*gene*	protein	Zv8 identifier	EBI identifier
*ptpn1*	PTP1b	ptpn1	FN428729
*ptpn2a*	tcPTPa	ptpn2	FN428730
*ptpn2b*	tcPTPb	ptpn2l	FN658836
*ptpn6*	shp1	ptpn6	FN428705
*ptpn11a*	shp2a	ptpn11	FN428738
*ptpn11b*	shp2b	zgc:63553	FN428707
*ptpn9a*	meg2a	LOC560176	FN428732
*ptpn9b*	meg2b	B8A4Y0_DANRE	FN428709
*ptpn12*	PEST	not found	not found
*ptpn18*	BDP1	zgc:113105	FN428701; FN428736
*ptpn22*	LyPTP	si:dkey-78k11.1	FN428715; FN428735
*ptpn3*	PTPh1	GENSCAN00000024273; Q9YHE7_DANRE	FN428731
*ptpn4a*	meg1a	ptpn4	FN428700; FN428714
*ptpn4b*	meg1b	si:rp71-1n18.2	FN658840
*ptpn21*	PTPd1	LOC799627	FN428703
*ptpn14*	PTP36	not found	not found
*ptpn13*	PTPBAS	A4QN87_DANRE	FN428711
*ptpn23a*	hdPTPa	PTPN23	FN428733
*ptpn23b*	hdPTPb	si:dkeyp-114f9.2	FN428734; FR668536
*ptpn20*	PTPTyp	GENSCAN00000028624; B8JK77_DANRE	FN428737
*ptpn5*	PTP-STEP	LOC559524	FN428702
*ptpn7*	HePTP	not found	not found

All candidate genes were aligned and assigned their respective gene names based on homology of the PTP domain and overall gene structure, and listed here are the non-receptor PTPs by gene name, protein name, Zv8 identifier and EBI accession number. Fragments of all genes were sequenced and respective sequences were submitted to the EMBL-EBI nucleotide sequence database.

**Table 2:** Classical receptor PTP genes in the zebrafish genome.

*gene*	protein	Zv8 identifier	EBI identifier
*ptprc*	CD45	ptprc	FN428713
*ptpra*	RPTPá	ptpra	FN428716
*ptprea*	RPTPåa	LOC567443	FN653010
*ptpreb*	RPTPåb	NP_001038642.1	FN653011; FN653012
*ptprm*	RPTPì	GENSCAN00000005834; Z6923F	FN428719
*ptprk*	RPTPê	ptprk	FN428708
*ptprt*	RPTPñ	GENSCAN00000013375; zc137e24.za	FN428710
*ptprua*	RPTPëa	GENSCAN00000035737; A3QK35_DANRE	FN428712; FN665786
*ptprub*	RPTPëb	GENSCAN00000026767; ptpru	FN665787
*ptprfa*	LARa	ptprf	FN428740
*ptprfb*	LARb	si:dkey-21k10.1	FN428739
*ptprsa*	RPTPóa	B8JLS9_DANRE	FN428741
*ptprsb*	RPTPób	PTPRS	FN428742
*ptprda*	RPTPäa	ptprd	FN428718
*ptprdb*	RPTPäb	zgc:165626	FN428717
*ptprga*	RPTPãa	PTPRG	FN428720
*ptprgb*	RPTPãb	ca16b	FN653013; FN653014
*ptprza*	RPTPæa	PTPRZ1	FN658837; FN658838
*ptprzb*	RPTPæb	wu:fc63b11	FN428721
*ptprb*	RPTPâ	PTPRB	FN428722
*ptprja*	dep1a	ptprja	FN428723
*ptprjb*	dep1b	GENSCAN00000023903; LOC100006189	FN428724
*ptprh*	sap1	PTPRH	FN428725
*ptprq*	PTPS31	A8DZA4_DANRE	FN428726
*ptpro*	GLEPP	ptpro	FN428727
*ptprr*	pcPTP	Zv7:ENSDARG00000068023; GENSCAN00000030970; Zv8_NA3250.7	FN658839
*ptprna*	IA2a	LOC564351	FN428704
*ptprnb*	IA2b	ptprn	FN428728
*ptprn2*	IA2â	ptprn2	FN428706

All candidate genes were aligned and assigned their respective gene names based on homology of the PTP domain and overall gene structure, and listed here are the receptor type PTPs by gene name, protein name, Zv8 identifier and EBI accession number. Fragments of all genes were sequenced and respective sequences were submitted to the EMBL-EBI nucleotide sequence database.

**Figure 2:**
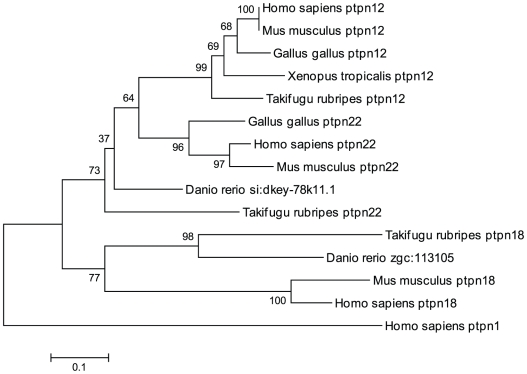
Alignment of truncated PTP domains of the NT4 non-receptor class. The PTP domains of genes of the NT4 class from several species were truncated to approximately correspond to the limited available sequence from si:dkey-78k11.1. The evolutionary history was inferred using the Neighbor-Joining method. The optimal tree with the sum of branch length = 3.38256318 is shown. The percentage of replicate trees in which the associated taxa clustered together in the bootstrap test (1000 replicates) are shown next to the branches. The tree is drawn to scale, with branch lengths in the same units as those of the evolutionary distances used to infer the phylogenetic tree. The evolutionary distances were computed using the Dayhoff matrix based method and are in the units of the number of amino acid substitutions per site. All positions containing alignment gaps and missing data were eliminated only in pairwise sequence comparisons (Pairwise deletion option). There were a total of 109 positions in the final dataset. Phylogenetic analyses were conducted in MEGA4.

Our search for candidate genes of the three non-receptor NT4 subtype genes, *ptpn12*, *ptpn18* and *ptpn22*, resulted in two candidates, zgc:113105 and si:dkey-78k11.1. The former clearly aligns with *ptpn18*, but the latter is heavily truncated with only limited predicted sequence information in the PTP domain. The truncated part of the PTP domain aligns with *ptpn22* in our phylogenetic tree (Fig. 1), and to ensure that this is a member of the NT4 class PTPs, we derived a phylogenetic tree with truncated PTP domains from several species corresponding to the available sequence in the zebrafish genome (Fig. 2). When aligned with truncated counterparts, si:dkey-78k11.1 still aligns with *ptpn22* and based on sequence homology, we conclude that this predicted gene encodes a fragment of zebrafish *ptpn22* and the missing part of the gene is probably encoded by a poorly sequenced part of the genome.

### Spatio-temporal expression of PTPs in zebrafish embryos

**Figure 3:**
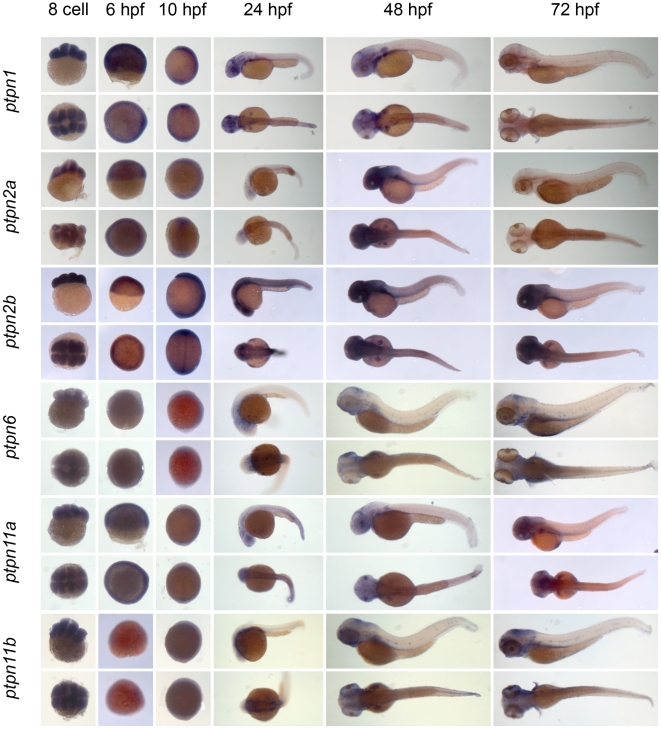
Expression patterns of the classical PTPs during zebrafish development. Albino zebrafish embryos were fixed at the following stages: 8 cell, 6 hpf, 10 hpf, 1 dpf, 2 dpf and 3 dpf. Whole mount *in situ* hybridization experiments were done with probes generated to target the classical PTPs and pictures were taken. Shown are 8 cell stage; top: lateral view, animal pole on top; bottom: animal pole view. 6 hpf; top: lateral view, animal pole on top, bottom: animal pole view. 10 hpf; top: lateral view, dorsal to the right, bottom: dorsal view anterior towards the top. 24, 48 and 72 hpf; top: lateral view anterior to the left, bottom: dorsal view anterior to the left.

**Figure 4:**
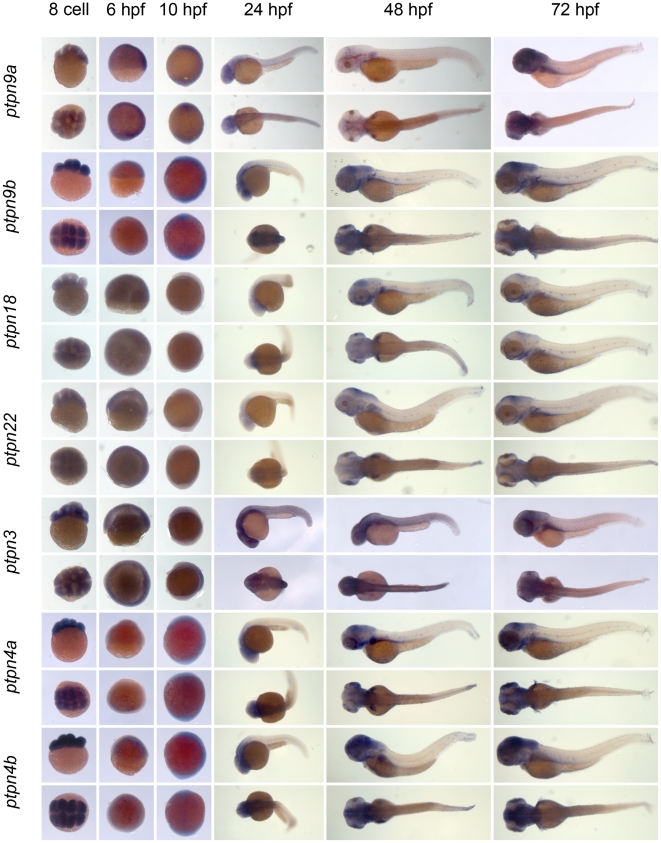
See legend to Fig. 3.

**Figure 5:**
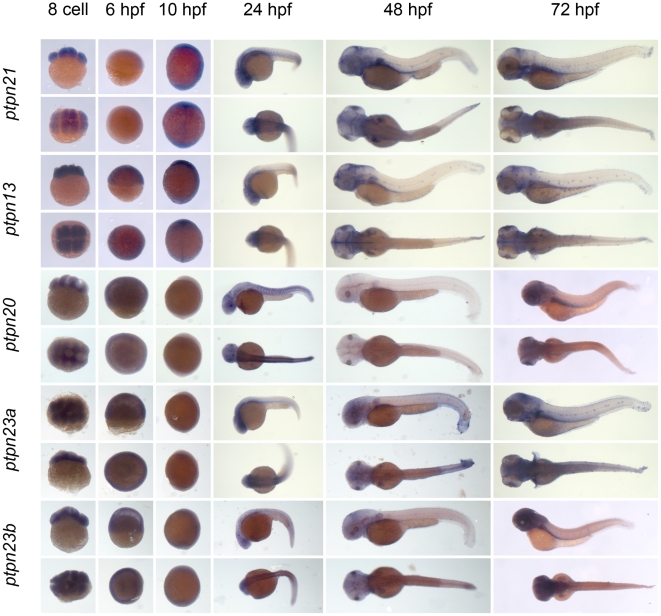
See legend to Fig. 3.

**Figure 6:**
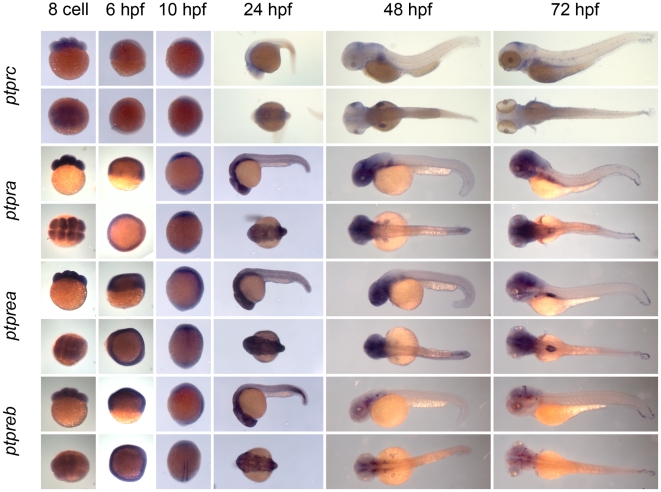
See legend to Fig. 3.

**Figure 7:**
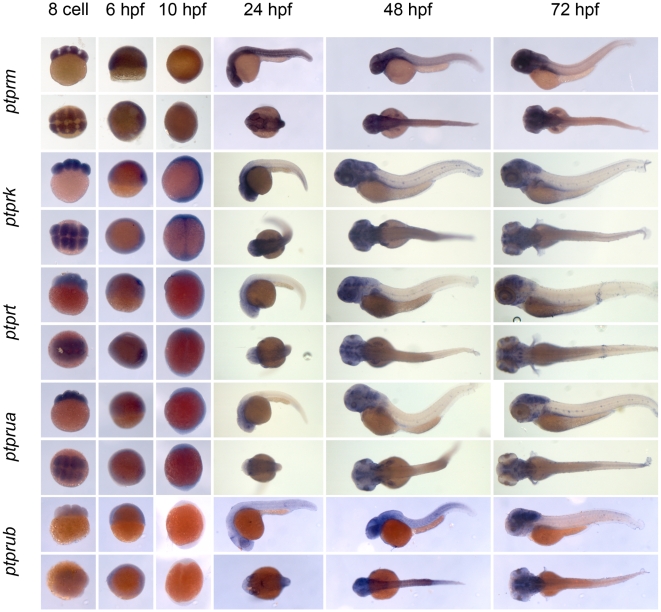
See legend to Fig. 3.

**Figure 8:**
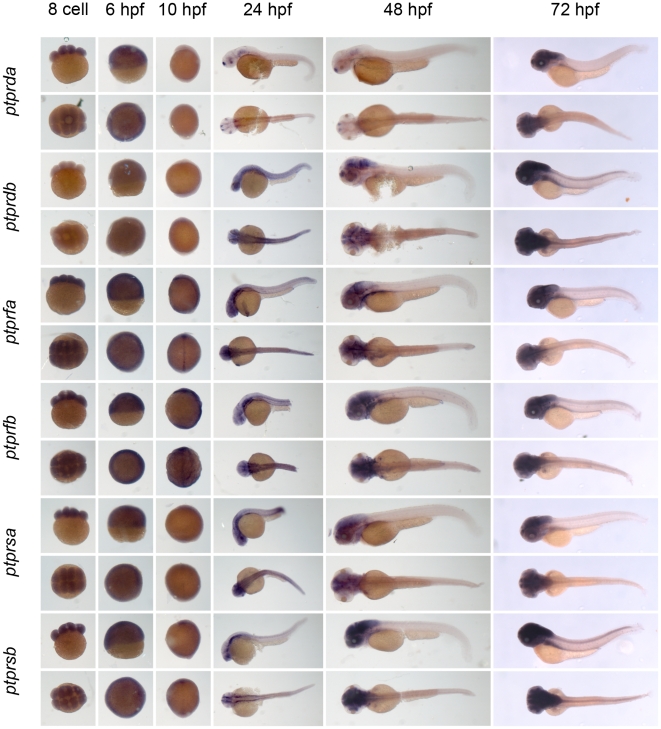
See legend to Fig. 3.

**Figure 9:**
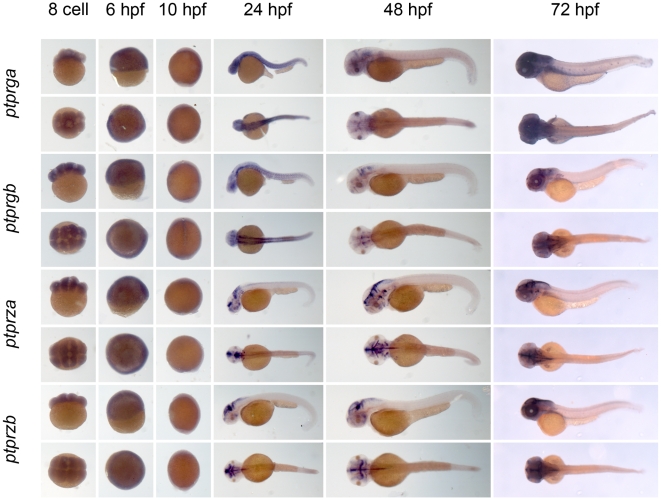
See legend to Fig. 3.

**Figure 10:**
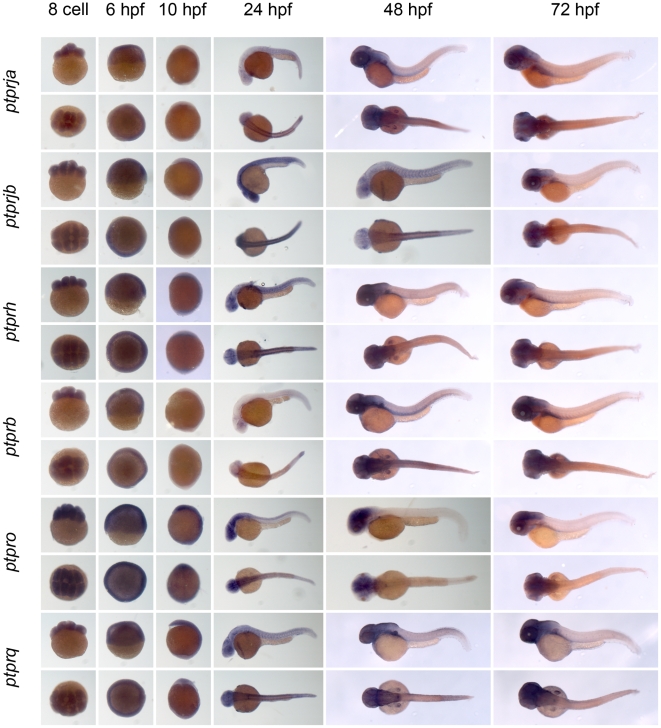
See legend to Fig. 3.

**Figure 11:**
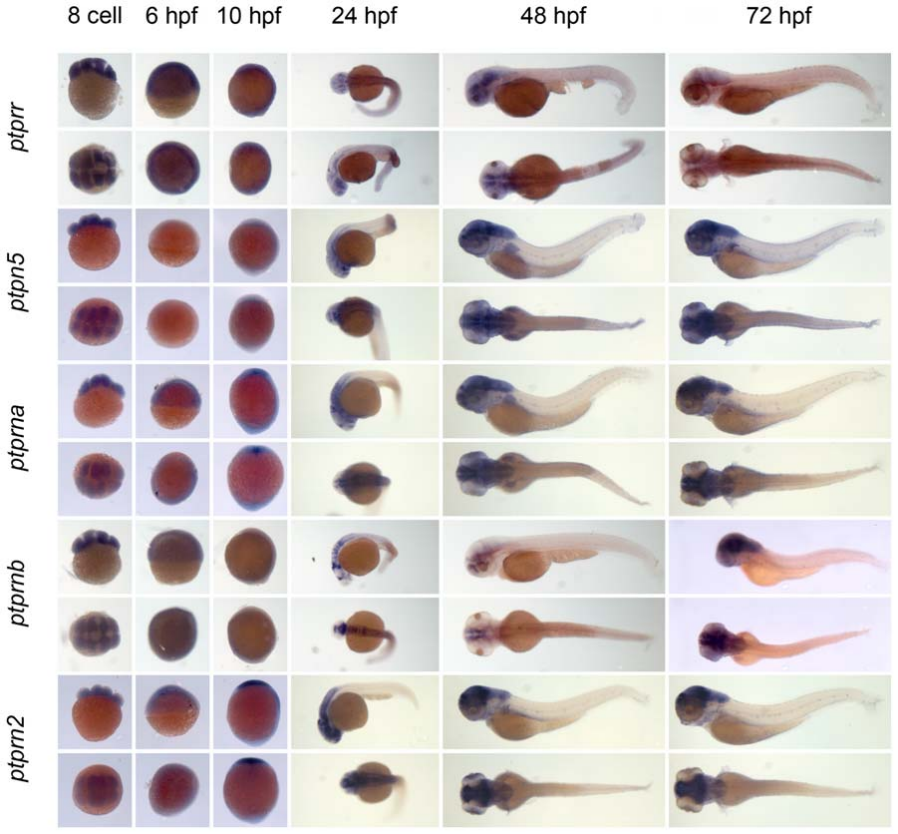
See legend to Fig. 3.

To investigate whether the genes we identified *in silico* are actually expressed in the zebrafish we amplified fragments of all PTP genes by RT-PCR. Based on our sequence information, we designed primer sets spanning approximately 800 bp coding region for every PTP encoding gene that we identified in the zebrafish genome, as well as a nested primer set with a T7 primer in the reverse oligo, facilitating sequencing and probe generation (Table S1). RNA was isolated from a mixture of 1 dpf and 2 dpf zebrafish embryos and RT-PCR was done using oligo dT priming and the two sets of specific primers for each gene. The PCR products were sequenced and these sequences were verified by BLASTing. The sequences were submitted to the EMBL-EBI database and accession numbers are shown in Table 1 and **2** **. Using this approach, all 48 PTP encoding genes that we identified** ***in silico*** **were verified by RT-PCR and sequencing.** As a first step to assess the function of the genes encoding the family of classical PTPs, we characterized the expression patterns at six distinct stages of zebrafish development by *in situ* hybridization. Using the T7 tag in the reverse nested oligo we generated antisense DIG-labeled RNA probes for whole mount *in situ* hybridization. Albino zebrafish embryos were obtained and fixed at the 8 cell stage, 6 hpf (shield stage), 10 hpf (1 somite stage), 24 hpf, 48 hpf and 72 hpf. Whole mount *in situ* hybridization experiments were done as described in the Materials and Methods section. All DIG labeled antisense probes gave staining patterns in at least one of the time points taken (Fig. 3, 4, 5, 6, 7, 8, 9, 10 and 11), indicating that every PTP encoding gene is expressed in zebrafish embryos.

We proceeded to analyze the expression data of all PTP genes (Fig. 3, 4, 5, 6, 7, 8, 9, 10 and 11) and summarized these in Tables 3 and 4. We conclude that almost every PTP is maternally provided (8 cell stage) and most PTPs are expressed ubiquitously at early stages (6 hpf and 10 hpf). At later stages expression patterns start to differentiate for individual genes, with most genes giving staining in specific tissues or organs as opposed to the ubiquitous expression seen at earlier stages.

**Table 3:** Expression patterns of zebrafish non-receptor PTPs.

PTP type:	*gene:*	8 cell	6 hpf	10 hpf	24 hpf	48 hpf	72 hpf
NT1	*ptpn1*	++	++	++	s	s	−
	*ptpn2a*	+	+	+	+/−	+	−
	*ptpn2b*	++	+	ss	ss	++	++
NT2	*ptpn6*	+/−	+/−	−	+/−	+/−	s
	*ptpn11a*	+	+	+	s	s	s
	*ptpn11b*	++	−	+/−	s	s	s
NT3	*ptpn9a*	+/−	+	+	s	+/−	+
	*ptpn9b*	++	+	++	++	ss	ss
NT4	*ptpn18*	+/−	+/−	−	+/−	s	s
	*ptpn22*	+/−	+/−	−	−	s	s
NT5	*ptpn3*	+	++	+	+	+	+
	*ptpn4a*	++	−	+/−	+	s	ss
	*ptpn4b*	++	−	s	+	ss	ss
NT6	*ptpn21*	+	−	s	s	s	s
NT7	*ptpn13*	++	+	s	s	s	s
NT8	*ptpn23a*	+	+	−	s	s	ss
	*ptpn23b*	+	+	+/−	s	s	s
NT9	*ptpn20*	+	+	−	s	+/−	s
R7	*ptpn5*	+	−	−	s	s	ss

Expression patterns of all classical non-receptor PTPs at 6 distinct stages of zebrafish development as shown in Fig. 3, 4 and 5 were analyzed and quantified as “−” for no expression, “+/−” for faint expression, “+” for expression and “++” for strong expression, “s” for localized expression or “ss” for strong localized expression.

**Table 4:** Expression patterns of zebrafish receptor PTPs.

PTP type:	*gene:*	8 cell	6 hpf	10 hpf	24 hpf	48 hpf	72 hpf
R1/R6	*ptprc*	+/−	+/−	s	s	s	s
R4	*ptpra*	++	+	+	s	s	s
	*ptprea*	++	++	s	s	s	s
	*ptpreb*	+	++	s	s	s	s
R2B	*ptprm*	++	++		ss	ss	ss
	*ptprk*	++	+	s	ss	ss	ss
	*ptprt*	+	+/−	+/−	s	ss	ss
	*ptprua*	++	+/−	+	+	ss	ss
	*ptprub*	−	+/−	−	+	ss	ss
R2A	*ptprfa*	+	++	s	ss	ss	s
	*ptprfb*	+	++	s	s	s	s
	*ptprsa*	+	+	+/−	s	s	ss
	*ptprsb*	+	+	+/−	s	ss	++
	*ptprda*	+/−	+	−	s	s	s
	*ptprdb*	−	+/−	−	ss	ss	ss
R5	*ptprga*	+/−	+	−	ss	s	++
	*ptprgb*	+	+	s	s	s	s
	*ptprza*	+	+	−	s	ss	ss
	*ptprzb*	+/−	+	−	s	ss	ss
R3	*ptprb*	+/−	+	−	+/−	ss	s
	*ptprja*	+/−	+/−	−	s	ss	s
	*ptprjb*	+	+	−	ss	s	s
	*ptprh*	+	+	+	ss	s	s
	*ptpro*	++	++	s	s	s	s
	*ptprq*	+	+	+	s	+	++
R7	*ptprr*	++	++	++	s	s	s
R8	*ptprna*	+	+	s	ss	ss	ss
	*ptprnb*	+	+	−	s	s	s
	*ptprn2*	+/−	+/−	s	ss	ss	ss

Expression patterns of all classical receptor PTPs at 6 distinct stages of zebrafish development as shown in Fig. 6, 7, 8, 9, 10 and 11 were analyzed and quantified as “−” for no expression, “+/−” for faint expression, “+” for expression and “++” for strong expression, “s” for localized expression or “ss” for strong localized expression.

### Expression patterns of duplicated genes

**Figure 12:**
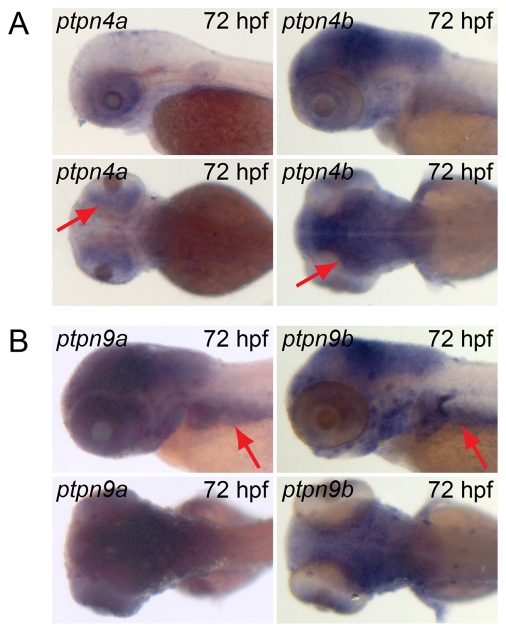
Similar and distinct expression patterns of duplicated non-receptor PTPs. Albino zebrafish embryos were fixed at 24 hpf and whole mount *in situ* hybridization was done with probes generated to target (A) *ptpn4a* and *ptpn4b* or (B) *ptpn9a* and *ptpn9b*. Depicted are (top) lateral view and (bottom) dorsal view of 72 hpf embryos.

Fourteen genes encoding PTPs are duplicated in the zebrafish genome and we investigated the expression patterns of the duplicated genes in detail. Five of the non-receptor PTPs are duplicated in the zebrafish genome, *ptpn2*, *ptpn4*, *ptpn9*, *ptpn11* and *ptpn23*. The orthologue of *ptpn4* has been reported to be involved in establishment and maintenance of axon projections in the central brain in *Drosophila* [26]. Analysis of the expression pattern in zebrafish shows that *ptpn4b* is expressed in the fore-, mid- and hindbrain at 3 dpf (Fig. 12A, arrows) and at earlier stages (Fig. 4), which is consistent with the expression pattern in *Drosophila*. *Ptpn4a*, however, is expressed in the retina exclusively (Fig. 12A, arrows). Hence, despite high homology of ptpn4a and ptpn4b, their expression patterns are mutually exclusive. *Ptpn9* has been reported to be involved in platelet and lymphocyte activation through vesicle trafficking [27], [28], but has also been shown to be an antagonist of hepatic insulin signaling in mice [29]. In zebrafish the expression patterns of *ptpn9a* and *ptpn9b* appear very similar, with expression in the brain and liver at 3 dpf (Fig. 12B, arrows).

**Figure 13:**
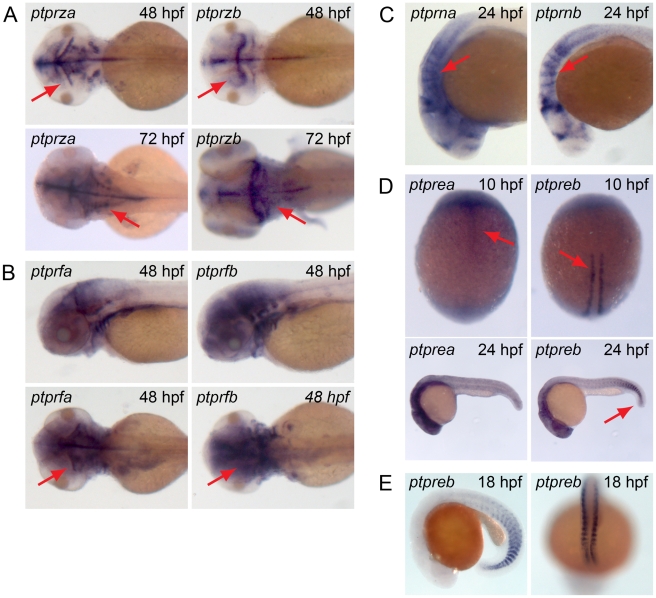
Overlapping and mutually exclusive expression patterns of duplicated receptor PTPs. Albino zebrafish embryos were fixed at different stages and whole mount *in situ* experiments were done with probes generated to target (A) *ptprza* and *ptprzb*, (B) *ptprfa* and *ptprfb*, (C) *ptprna* and *ptprnb*, (D) *ptprea* and *ptpreb* and (E) *ptpreb*. Depicted are (A) dorsal views of (top) 48 hpf embryos and (bottom) 72 hpf embryos, (B) (top) lateral views and (bottom) dorsal views of 48 hpf embryos, (C) lateral views of 24 hpf embryos and (D) dorsal view of 10 hpf embryos (top), lateral view of 24 hpf embryos (bottom). (E) Lateral view (left) and dorsal view (right) of the tip of the tail of 18 hpf embryos.

Several receptor PTPs are duplicated in the zebrafish genome, including the entire R5 type and R2B type PTPs. *Ptprz* has been reported to have a function in memory by dephosphorylating p190RhoGAP in the hippocampus [30]. In the zebrafish *ptprza* and *ptprzb* stain specific, but very distinct structures in the brain (Fig. 13A). *Ptprza* is expressed in the hindbrain and in more anterior parts of the tectum, whereas *ptprzb* is expressed in the hindbrain and the posterior tectum. *Ptprf* plays a role in the development and maintenance of excitatory synapses and axon guidance in cultured rat hippocampal neurons [31]. In the zebrafish *ptprfa* and *ptprfb* show very similar expression patterns and they are expressed mainly in the central nervous system and the liver (Fig. 13B). *Ptprn* is a catalytically inactive PTP predominantly expressed in neuroendocrine cells that possess regulated secretory granules [32], [33]. Expression in the zebrafish at 24 hpf is limited to rhombomeres and is similar for *ptprna* and *ptprnb* (Fig. 13C). *Ptpre* plays a role in osteoclast bone adhesion and resorption in mammals and in convergence and extension cell movements during zebrafish gastrulation [19], [34]. In the zebrafish *ptprea* is expressed in anterior parts of the notochord, whereas *ptpreb* is expressed in the more posterior adaxial cells at 10 hpf (Fig. 13D). At 24 hpf *ptpreb* is expressed at the tip of the tail and in the brain, whereas *ptprea* expression is restricted to the brain. At later time points, *ptpreb* expression is fading, while *ptprea* remains strongly expressed in the entire brain (Fig. 13D). The *ptpreb* expression pattern in somites in the tail is reminiscent of expression patterns of genes that are expressed in newly formed somites. Therefore, we analyzed expression of *ptpreb* at 18 hpf and indeed found strong expression of *ptpreb* in newly formed somites, indicating that *ptpreb* is expressed during somitogenesis (Fig. 13E). We have done functional assays on *ptpre* in the zebrafish previously, but focused on double knockdowns of both *ptprea* and *ptpreb* [19]. The function of the individual *ptpre* genes resulting from their diverging expression patterns remains to be determined.

Analysis of the expression of duplicated genes reveals that the expression pattern of approximately half of them is similar, whereas the other half has distinct or even mutually exclusive expression patterns. Similar expression patterns may suggest redundancy among the duplicated genes and divergence of the expression patterns of duplicated genes suggests a non-redundant function. Similarities and differences in the functions of the duplicated PTP genes remain to be determined.

According to the duplication-degeneration-complementation (DDC) model [35] degenerative mutations in regulatory elements can increase the probability of duplicate gene preservation and the mechanism of preservation of duplicate genes is partitioning of ancestral functions. The teleost genome is believed to have been duplicated about 320 million years ago [36], [37]. If only random mutations in genes are considered, most genes would have disappeared by now. There are 37 PTP genes present in the mammalian genome, of which 14 genes are duplicated in the zebrafish genome, ammounting to 39% of all PTP genes. In half of these cases we observed that expression patterns are complementary between duplicated genes, in accordance with the DDC model. The other half of duplicated genes shows expression patterns that are similar. However, expression patterns may be complementary between duplicated genes on the cellular or subcellular level, at which resolution we cannot distinguish with our current data. It would be interesting to further investigate the function of duplicated genes with seemingly identical expression patterns.

In conclusion, we have identified all genes in the zebrafish genome that encode classical PTPs. We identified a second copy of *ptpn23* which appears to lack a PTP domain, which is interesting because whereas HD-PTP itself does not exhibit PTP activity, its PTP domain is required for its function and hence *ptpn23b* may have lost its PTP domain in the course of evolution. We have established that all PTP genes are expressed by RT-PCR and we confirmed partial sequences of these genes. Moreover, we have established the spatio-temporal expression patterns of all genes encoding classical PTPs in zebrafish embryos, which is a first step towards understanding their function. Whereas some duplicated genes have largely overlapping expression patterns, others are distinct or even mutually exclusive, which suggests that the function of the latter group has diverged since their duplication.

## Materials and Methods

### Bioinformatics

The Ensembl database (http://www.ensembl.org/Multi/blastview/) was used to BLAST human PTP domains against the latest version of the zebrafish genome (Zv8) or other genomes. TBLASTN algorithm was used against the LATESTGP DNA database, using “near exact matches” for search sensitivity. To identify PTP domain protein sequences in human and zebrafish proteins we used Prosite (http://www.expasy.org/prosite/). Human and zebrafish PTP domains were aligned for generating a phylogenetic tree using MEGA4 [38] software (http://www.megasoftware.net/). Aligning was done by clustalW using the PAM matrix. Phylogenetic tree construction was done using Neighbor-Joining, bootstrapped tree inference and using the Dayhoff Matrix (Amino Acid) as a model, pairwise deletion for gaps, homogenous pattern among lineages, uniform rates among sites and all substitutions included.

### Whole mount *in situ* hybridization

Wild type zebrafish were kept and embryos were raised under standard conditions at the Hubrecht Institute. Only wild type embryos up to 3 dpf were used for these experiments, which does not require approval of the animal experiments committee according to national and European law. Zebrafish embryos of the appropriate stages were collected and fixed in PBS containing 4% PFA. Embryos were dechorionated when needed and transferred to 100% MeOH at −20°C for at least 12 hours. Whole mount *in situ* hybridization was performed as described before [39].

### Accession numbers

All *in silico* identified zebrafish genes were verified by sequencing and sequences were submitted to EMBL-EBI nucleotide database. All accession numbers are shown in Tables 1 and 2.

## Supporting Information

Table S1 Oligos used for probe generation and sequencing. Listed are all oligos used for the generation of in situ probes and to sequence cDNA. Oligo 1 and 4 serve as forward and reverse primer, respectively. Oligos 2 and 3 are nested forward and reverse primers. Oligo 3 contains a T7 tag, which facilitates generation of antisense probes. All probes were designed to span approximately 800 bp of known coding sequence. n.d., not done.
(0.07 MB DOC)
