# Supplementary material for: Identification and Expression of the Family of Classical Protein-Tyrosine Phosphatases in Zebrafish
Source: PLoS One. 2010 Sep 3;5(9):e12573. doi: 10.1371/journal.pone.0012573 (PMC2933243; doi:10.1371/journal.pone.0012573)
Supplement: Table S1 — Oligos used for probe generation and sequencing. Listed are all oligos used for the generation of in situ probes and to sequence cDNA. Oligo 1 and 4 serve as forward and reverse primer, respectively. Oligos 2 and 3 are nested forward and reverse primers. Oligo 3 contains a T7 tag, which facilitates generation of antisense probes. All probes were designed to span approximately 800 bp of known coding sequence. n.d., not done. (0.07 MB DOC) [file pone.0012573.s001.doc]

*Table S1.* ***Oligos used for probe generation and sequencing.***

| **gene** | **oligo 1** | **oligo 2** | **oligo 3** | **oligo 4** |
| --- | --- | --- | --- | --- |
| *ptpn1* | gagtttcgggaaatcgatga | tccagtgacctaccatgcaa | acgtaatacgactcactatagggggcggaggagttagttcagg | ttgtgatgctctcggtgaag |
| *ptpn2a* | gtggcaaagttcccagaaaa | atgatcacagtcgggtgaaa | acgtaatacgactcactatagggtgctcttcctttggaaatcg | ttttctgcacttgctgtgct |
| *ptpn2b* | tcaaagtggccaagtatcca | gcctggtggtaatggaagaa | acgtaatacgactcactatagggtctttgagtcgatcccatcc | gtgttccagcatccctgtct |
| *ptpn3* | atccccactgtccactcatc | atccccactgtccactcatc | acgtaatacgactcactatagggacacgatgtctgaaccagca | gcctcacacacaaaggtgaa |
| *ptpn4a* | ccgctacagagacatctcacc | tctcaccttatgatgcaactcg | acgtaatacgactcactatagggagtatggcctcgcacacaa | ggccctcctcgtaaactttc |
| *ptpn4b* | ctcaatgaaggcgatcaggt | gcgaggaccaatcaggagag | acgtaatacgactcactatagggcacatcgctgtctccattgt | cgctggtctctcatggttct |
| *ptpn5* | cagctggagcttttctcctg | tcctgctctacacactgtctcaa | acgtaatacgactcactatagggactgcagtggacgatgacag | agcaatgaaacagccagtgc |
| *ptpn7* | n.d. | n.d. | n.d. | n.d. |
| *ptpn6* | atggttacgaacccccaga | gttgcctcgcaacaactgta | acgtaatacgactcactataggggacgtctttctttccctttgc | cagatccaccacccttcttc |
| *ptpn11a* | ggagctacattgccacacaa | cctgcagaacaccatcagtg | acgtaatacgactcactatagggagggctctgatctcctccac | agagctgtcgtccctcatgt |
| *ptpn11b* | aggacgtcatatggatgaagg | gctactcagggctgccttc | acgtaatacgactcactatagggtcctggtgttactgctggtg | cgtggcttatctcctggtgt |
| *ptpn9a* | gacgtccacaactggaacct | tcctgtggatgagcttgttg | acgtaatacgactcactatagggccgtctggcaaatgttaagc | tcgggtgtttgtatgctgaa |
| *ptpn9b* | aatgtgccgaaccacagaat | ccgaaccacagaatgacagg | acgtaatacgactcactatagggccctacattcccctgtctga | tctggccagctcatgtagag |
| *ptpn12* | ctaatcgccatcaaggaagc | cggggagtttttgaaactga | acgtaatacgactcactataggggcacaatgcacctgttcttc | atgccctttaccagctcaaa |
| *ptpn18* | tgcaggaggaatgaaagaaaa | aaacttcgtcaagggagctg | acgtaatacgactcactatagggtcggtggaactgcattgat | caggggctaatgatgcagat |
| *ptpn22* | n.d. | n.d. | n.d. | n.d. |
| *ptpn13* | tctctgccgctggttaaagt | ttcctgacggtcactacacg | acgtaatacgactcactatagggtgttcatctgcgtctttgct | cagcgcagcacatacagaat |
| *ptpn20* | ggagctttccagtcttgcag | cctcgagcagctagaaaagc | acgtaatacgactcactatagggtgagactgaggatgacgtctg | cgctggagtctcatctcctt |
| *ptpn23a* | gcagctgagacagagtgctg | actttgagggctgcagtacg | acgtaatacgactcactatagggctgtctggctgacccttagc | ttggcggaattaaattttcc |
| *ptpn23b* | tgtggtcagctgcactttct | tgcctgtgacatggacagat | acgtaatacgactcactataggggggtttcaccaatgatgcac | ctcgctgtaaagggatgagg |
| *ptpn21* | cagctctgaacggtttgacc | accctgtccaggattcctct | acgtaatacgactcactatagggatctcggacaggatgaccac | tgttccagacaggcgatcat |
| *ptpn14* | n.d. | n.d. | n.d. | n.d. |
| *ptpra* | ttccgggaagaattcaatg | ctgccagtgtgtcccattc | acgtaatacgactcactatagggctgcatatcggtctgaacca | tccagcaaagcttggaaaat |
| *ptprea* | tcgtctatcaggctctgctg | atggagacacagagctggac | acgtaatacgactcactatagggtgaaacacatccagcaaacc | ccgctgcattcgtaaactct |
| *ptpreb* | atggatatgatgcacgctga | cgctgtcagctcattcagac | acgtaatacgactcactataggggacccgctctaagatgttgc | cacggtctgaaaaacatcca |
| *ptprc* | gcgaagagaccagaaaacca | ccagtccaagaaccgctatg | acgtaatacgactcactatagggtctctctgatggtgttgagca | tgctctcattgccttgtttc |
| *ptprm* | agtttcacttcaccggttgg | caccggatgcttcattgtta | acgtaatacgactcactatagggtagatgcggaagatcctgct | gcgttgtagatgcggaagat |
| *ptprk* | ggcctatagtcgtccactgc | ggacggaccggatgttac | acgtaatacgactcactatagggaccccaaatactggaactgc | cacctgcaggatcagtttga |
| *ptprt* | ggaaacacagcaatcccagt | cagcaatcccagtgtgtgaa | acgtaatacgactcactatagggtgtgaaaaacgtccacgatg | gtctccaccatgttggcttt |
| *ptprua* | cgtagtggcactttctgtgc | aagactctccgcaactccaa | acgtaatacgactcactatagggctgacgtttgaccacagtcg | aaaatgccgaacttcacacc |
| *ptprub* | ggtcaactcctcccagcat | cactgtgtgcagttcagctactt | acgtaatacgactcactatagggtctaggctggcccgatattc | atccaggtgccacagcttat |
| *ptprfa* | agttgttctcacccctgtgg | ggctaccgtaagcagaatgc | acgtaatacgactcactatagggatgcagctggcattgatgta | cagaagtcctcggtggtctc |
| *ptprfb* | gttcggcagttccagttcat | ttcttacgcagggtcaaagc | acgtaatacgactcactatagggtgtctttggcacaccttgtt | actgctccttggttttgtgg |
| *ptprsa* | caggctggaggaaaagtcac | tatcgaactcgccaccttct | acgtaatacgactcactatagggcctctccagatttgggaaca | ttatgcacctgtccgatgaa |
| *ptprsb* | caggtcagtctgctggacac | tctgcacaagaacggctcta | acgtaatacgactcactatagggctgcggcatgttgtattctg | tttgggactccttgttctgg |
| *ptprda* | gacaggctgcttcattgtga | cgtgactctaatgcgctctc | acgtaatacgactcactataggggccaatccgtgaactgaaac | aatgaacccttcgccagatt |
| *ptprdb* | gcgcagaggaactacatggt | tcagtacgtgttcatccacga | acgtaatacgactcactatagggctcagcatcttgacggtctg | agatactccaacccggctct |
| *ptprga* | ccttgatggaagccattctt | ggggcaaaacacgactagag | acgtaatacgactcactatagggggctggatccgttctcttta | catactgccgtttctgtcca |
| *ptprgb* | tgacgcactaatggaagcag | acgtacacgaacagcctcct | acgtaatacgactcactatagggactgctgacaaggctgagca | gcttttccagcgaatttgtc |
| *ptprza* | aggagcctcatgcaagaca | tccaatgccaaacaaaatga | acgtaatacgactcactataggggacgtccaccacactctcg | gtagcgcttgctcgtcttct |
| *ptprzb* | aaagagcacaaatgtccttgc | ccagacatgggtgtaccaga | acgtaatacgactcactatagggggtgctgaccaaactcagaa | tagcgcttgctcgtcttctt |
| *ptprja* | gcgatacattcctggctgtt | tgagctggaaacaatcatca | acgtaatacgactcactatagggtctgctggccagtattcctc | tccaatcctccaatgggata |
| *ptprjb* | cacaacagtcaaaggctgct | gccctggctattgaaaacaa | acgtaatacgactcactatagggcgaatgaggtcatacgcaca | gctgctgcgttctggtaaat |
| *ptprh* | ctggctgtcatgtgggatta | tgtggtggaggtggatgtta | acgtaatacgactcactatagggagagtccaggatgcactggt | agccattgcgttgacataga |
| *ptprb* | attctgaccgaccgactgac | tgcatcaacctctgcctgta | acgtaatacgactcactatagggaccatgtatgagcgatgcag | ggggttatctcgttcacagc |
| *ptprq* | gccaagccgagattattgag | aatcggccaatcagtaagaa | acgtaatacgactcactatagggccagagtgctctgatggaga | catcttctccagagcggagt |
| *ptpro* | ttcgaggcctacctcaaaga | gggcaaagactcagcctaca | acgtaatacgactcactatagggtcagcatcacctcagtctgc | ctggtgtgtgactgctgctt |
| *ptprr* | accatgagaagaacggcatc | tgctggttatctgcctcaca | acgtaatacgactcactatagggatgacgatgacagggcagt | ccctcttctcaggccagtaa |
| *ptprna* | ggctgctaagtcctctctgg | ctctgggcaggcaggact | acgtaatacgactcactatagggtcaggaccatgtcaatcagg | tgcagcaatatcgatctctttc |
| *ptprnb* | cagcacatggctgctaagtc | acgtcacgtgtcagcagtgt | acgtaatacgactcactatagggcggttcaggaccatgtcaat | tgatctcgaatgtgctccag |
| *ptprn2* | atggaggatcacctgcaaaa | tgcaaaataaggatcgtttgg | acgtaatacgactcactatagggttctgctacagccgtcagtg | tgaggcagagctttcaggat |
